# Supplementary figures and images for: Debulking of the Femoral Stem in a Primary Total Hip Joint Replacement: A Novel Method to Reduce Stress Shielding
Source: Bioengineering (Basel). 2024 Apr 18;11(4):393. doi: 10.3390/bioengineering11040393 (PMC11047840; doi:10.3390/bioengineering11040393)

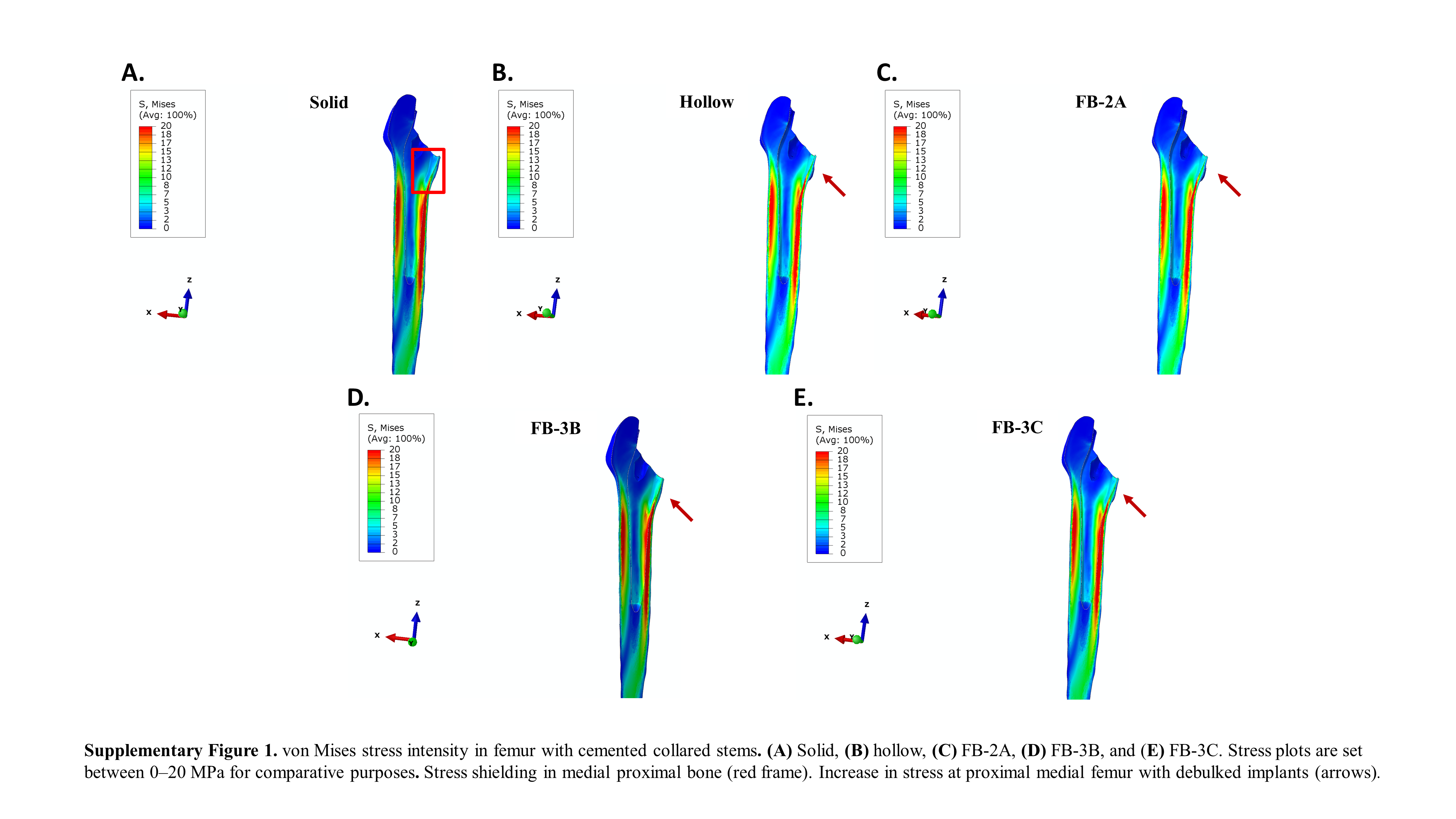

Supplement: Supplementary file 1 [file bioengineering-11-00393-s001.zip › Supplementary figures/Supplementary Figure 1.TIF]

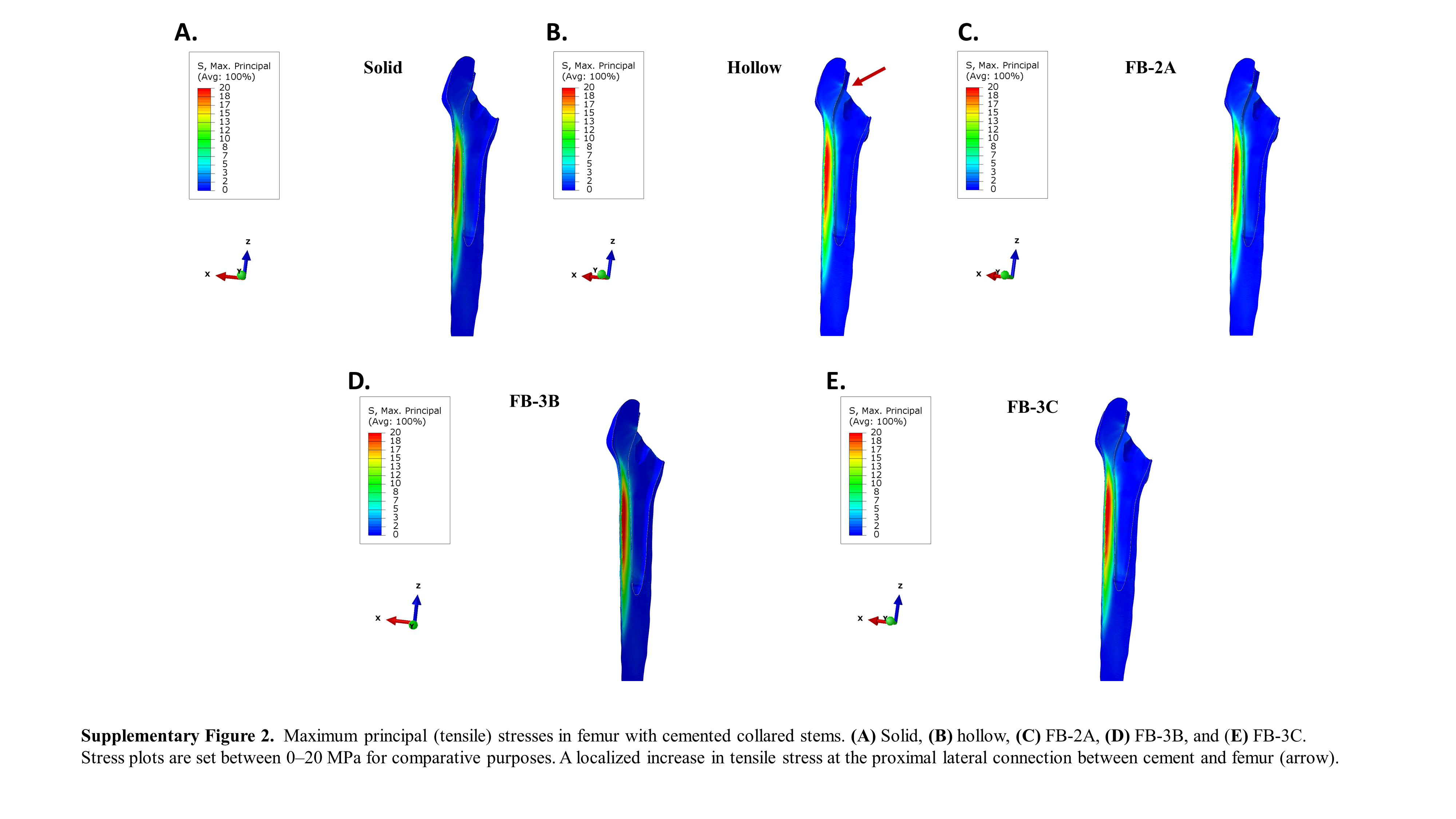

Supplement: Supplementary file 1 [file bioengineering-11-00393-s001.zip › Supplementary figures/Supplementary Figure 2.TIF]

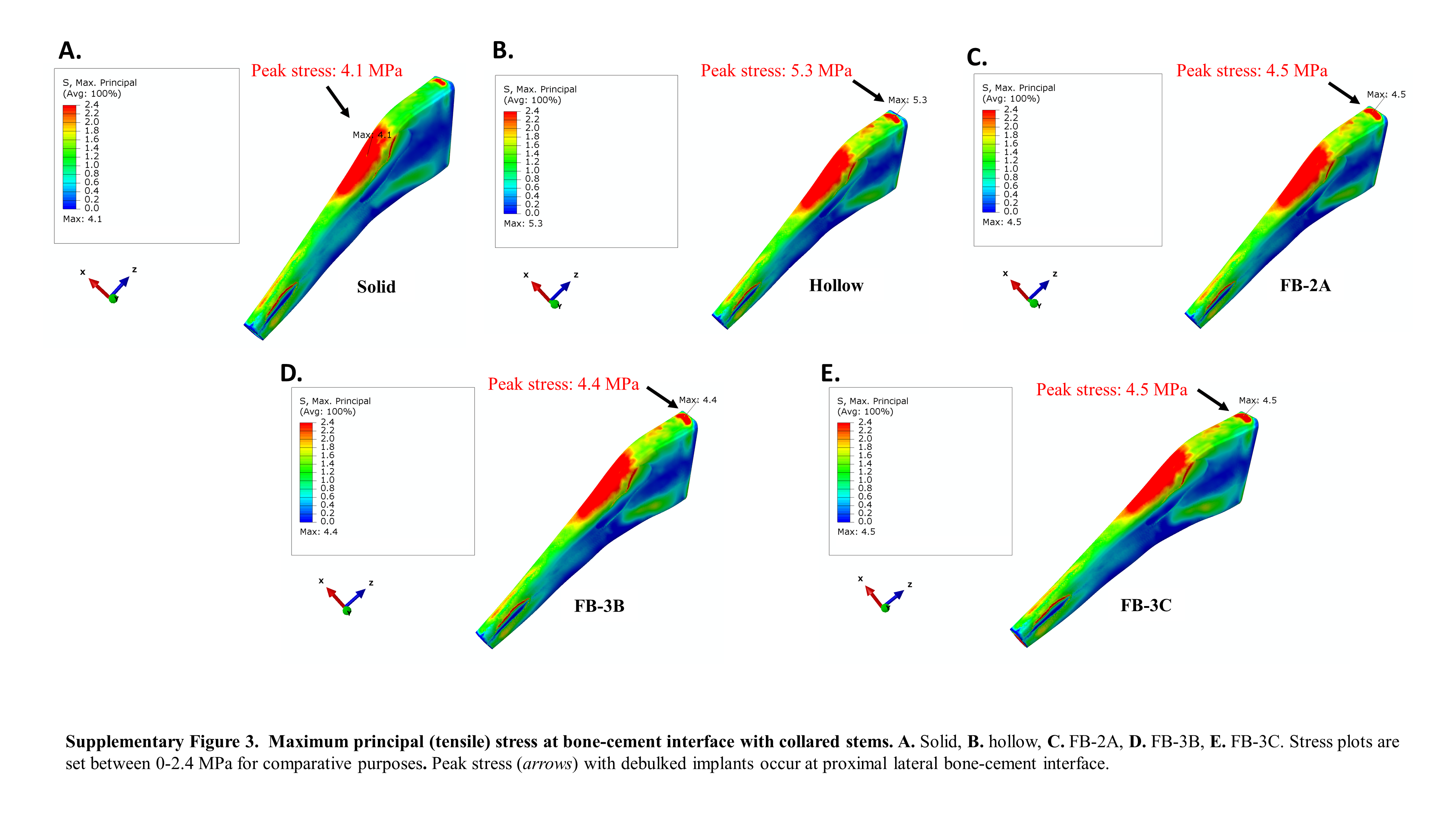

Supplement: Supplementary file 1 [file bioengineering-11-00393-s001.zip › Supplementary figures/Supplementary Figure 3.TIF]

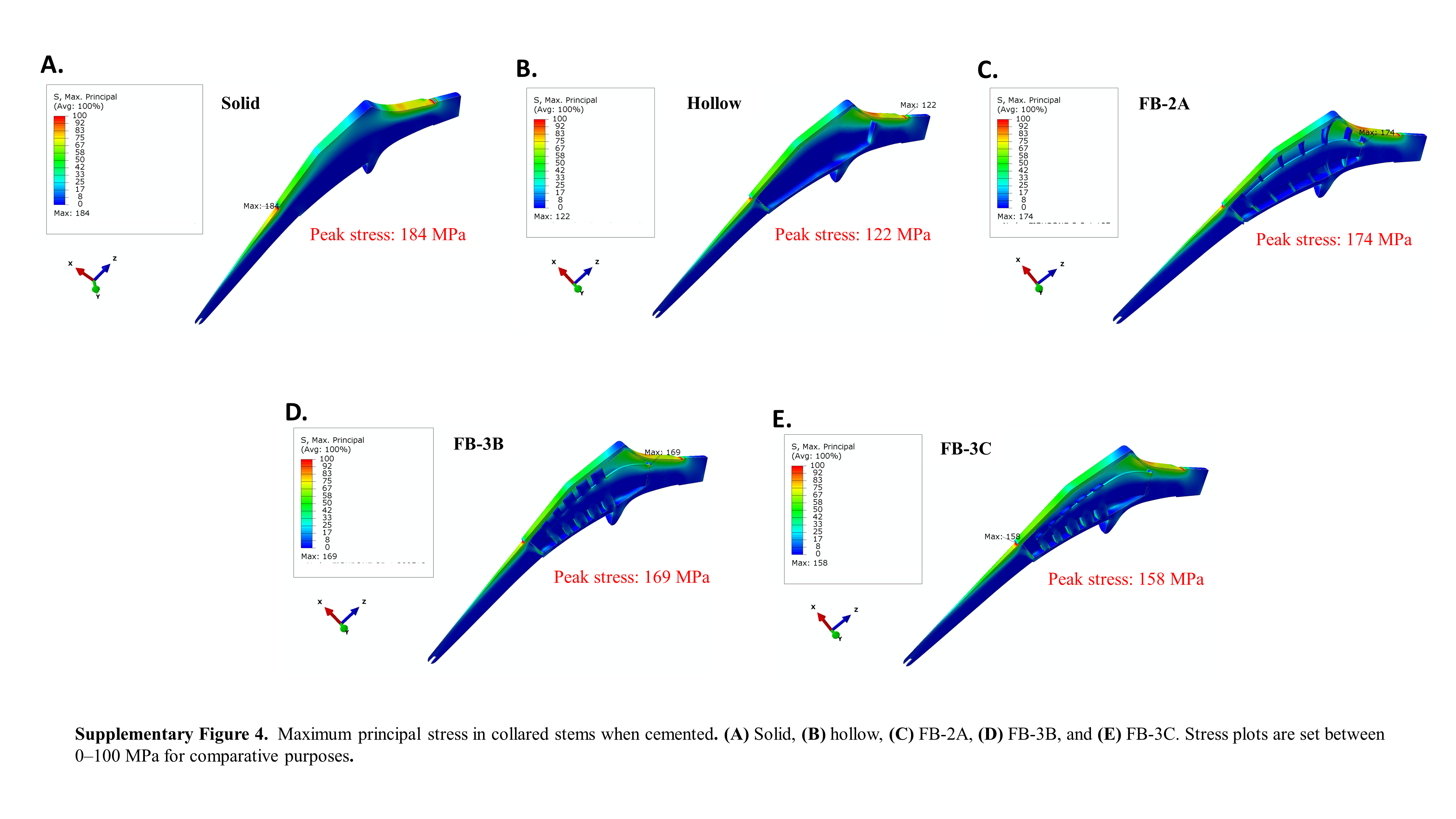

Supplement: Supplementary file 1 [file bioengineering-11-00393-s001.zip › Supplementary figures/Supplementary Figure 4.TIF]

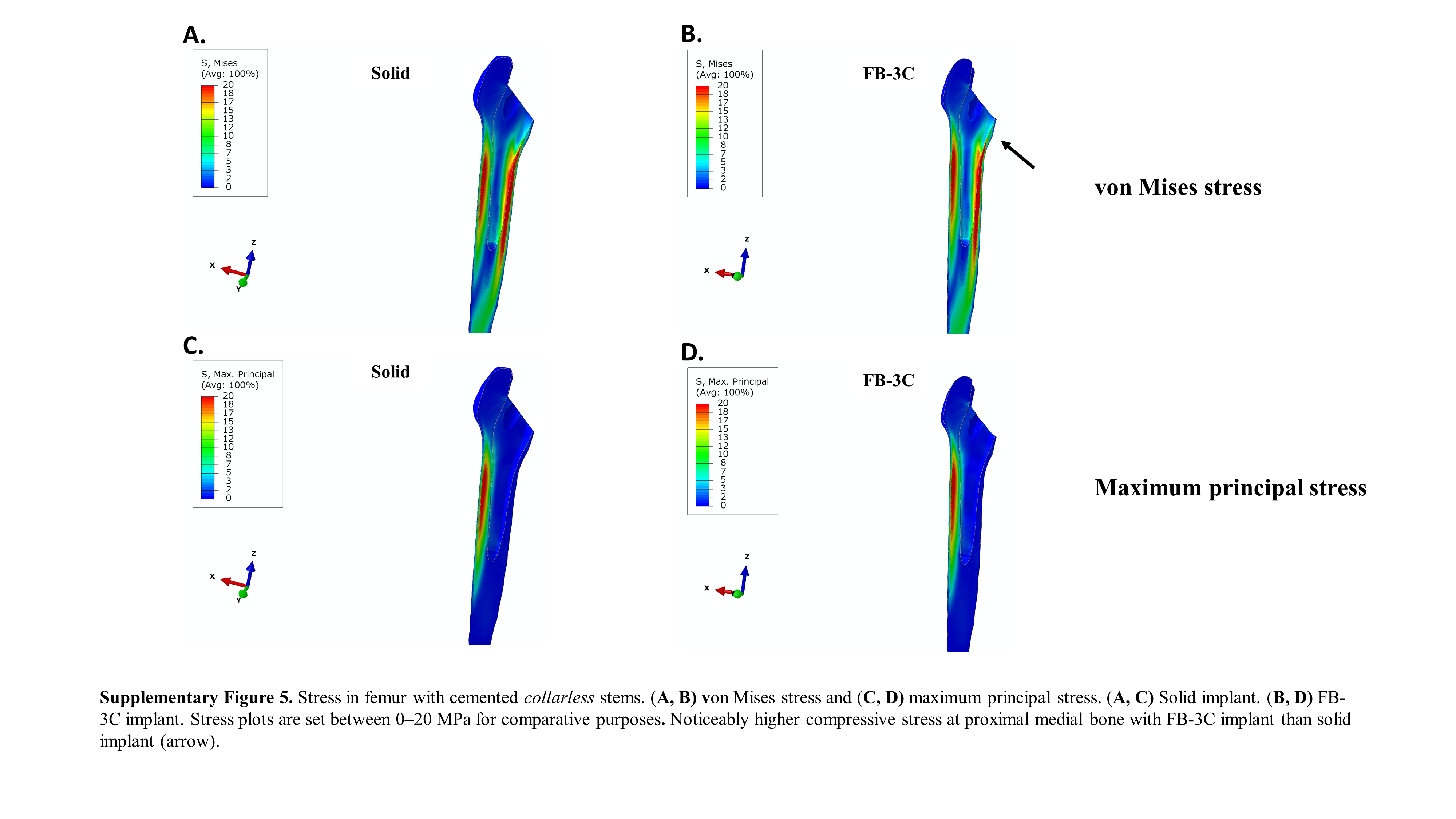

Supplement: Supplementary file 1 [file bioengineering-11-00393-s001.zip › Supplementary figures/Supplementary Figure 5.TIF]

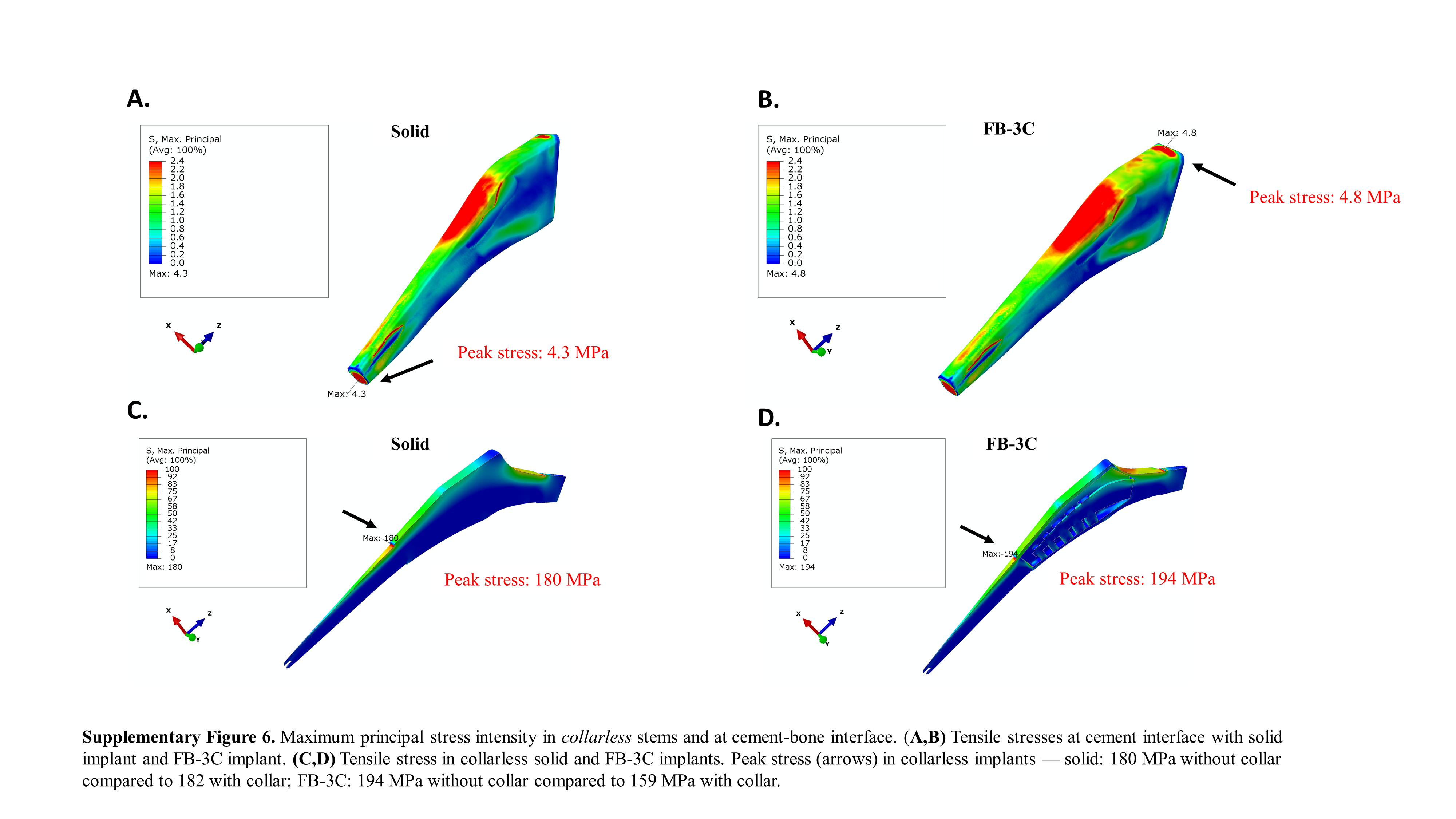

Supplement: Supplementary file 1 [file bioengineering-11-00393-s001.zip › Supplementary figures/Supplementary Figure 6.TIF]

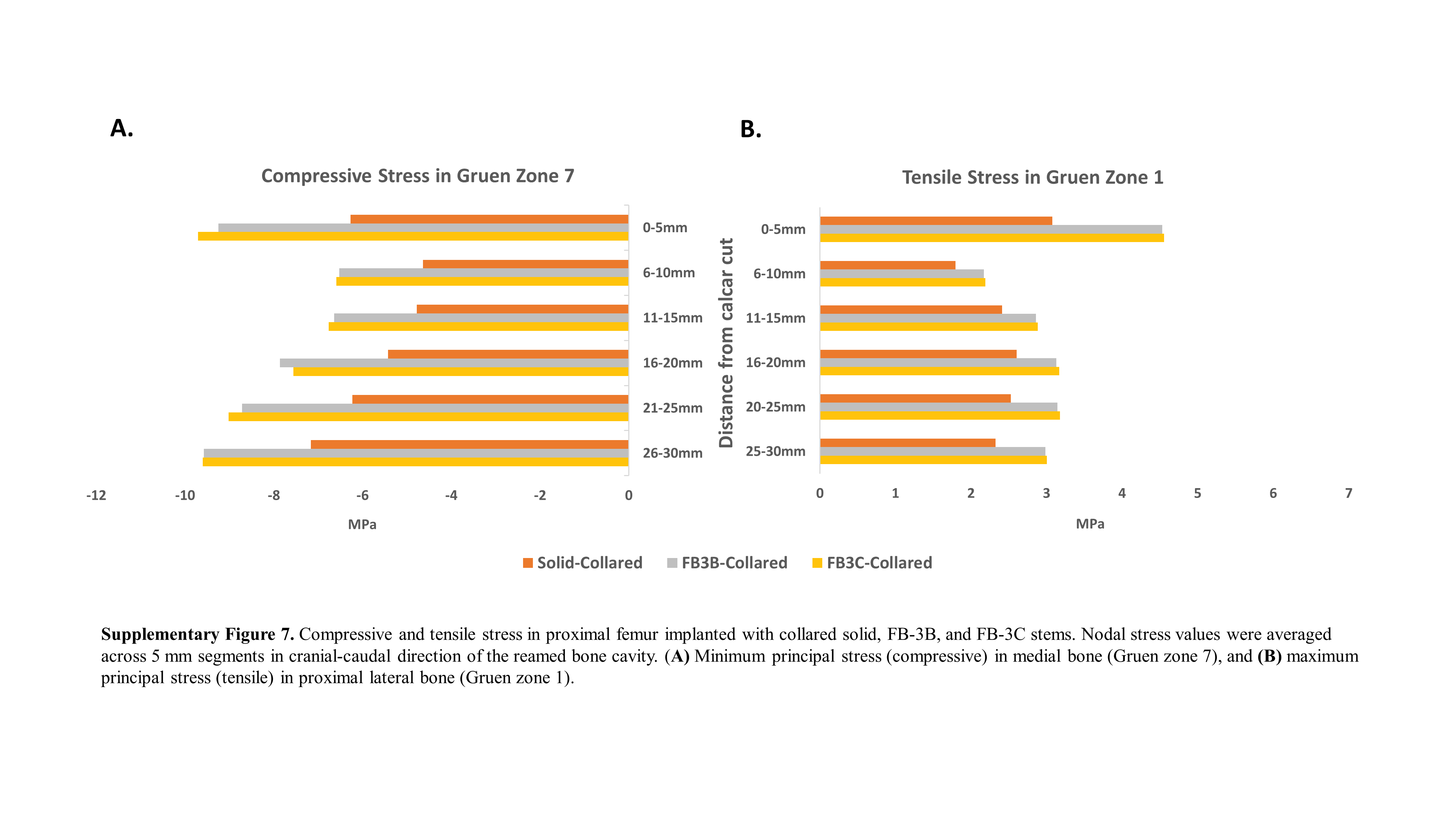

Supplement: Supplementary file 1 [file bioengineering-11-00393-s001.zip › Supplementary figures/Supplementary Figure 7.TIF]
